# PNET-PRISM: a multicenter-validated radiomics nomogram for noninvasive grading of pancreatic neuroendocrine tumors

**DOI:** 10.1186/s13244-026-02250-3

**Published:** 2026-03-24

**Authors:** Ying Li, Chengwei Chen, Mingzhi Lu, Jiajun Liu, Jieyu Yu, Danqun Zheng, Yilun Zheng, Yixuan Shen, Fang Liu, Tiegong Wang, Xu Fang, Jing Li, Jianping Lu, Chengwei Shao, Yun Bian

**Affiliations:** 1 Department of Radiology, Changhai Hospital, Shanghai, China; 2 Department of Radiation Oncology, Changhai Hospital, Shanghai, China; 3 Department of Nuclear Medicine, Ruijin Hospital, Shanghai Jiao Tong University School of Medicine, Shanghai, PR China

**Keywords:** Pancreatic neuroendocrine tumor, Computed tomography, Deep learning, Tumor grading, Artificial intelligence

## Abstract

**Background:**

Accurate preoperative grading of pancreatic neuroendocrine tumors (PNETs) is essential for optimal treatment selection, yet endoscopic ultrasound-guided fine-needle aspiration (EUS-FNA) yields inadequate tissue in up to 40% of cases and carries procedural risks, necessitating reliable noninvasive alternatives.

**Materials and methods:**

This multicenter retrospective study included 407 surgically confirmed PNET patients across training (*n* = 244), validation (*n* = 106), and external test (*n* = 57) cohorts. We developed a pancreatic radiomics integrated scoring model for PNET (PNET-PRISM), integrating multidimensional CT radiomics features from intratumoral, peritumoral, habitat, and deep learning domains using automated segmentation. A multidimensional deep learning radiomics score (M-DLR Score) was constructed from 13,542 features and combined with clinical variables for preoperative grade prediction.

**Results:**

PNET-PRISM demonstrated robust performance with AUCs of 0.92, 0.89, and 0.87 in training, validation, and external test sets, respectively, significantly outperforming clinical-only models (ΔAUC = 0.15–0.22, all *p* < 0.001). The model achieved perfect sensitivity (100%) in external validation and provided accurate grading in 13 of 25 patients (52%) where EUS-FNA yielded insufficient tissue. Net Reclassification Improvement analysis demonstrated significant improvement over clinical models across all datasets (NRI = 0.318–0.406, *p* ≤ 0.070). M-DLR Score stratification showed a significant association with progression-free survival (HR = 2.050, 95% CI: 1.484–2.833, *p* < 0.001).

**Conclusions:**

This validated radiomics-based nomogram serves as a powerful noninvasive decision-support tool for PNET risk stratification, effectively complementing EUS-FNA limitations and enabling optimized treatment pathways, particularly when biopsy is contraindicated or nondiagnostic.

**Critical relevance statement:**

This CT-based radiomics nomogram reliably grades pancreatic neuroendocrine tumors (PNETs) and predicts prognosis. This study addresses endoscopic ultrasound-guided fine-needle aspiration (EUS-FNA) limitations and advances clinical radiology by enabling safer triage and personalized management when tissue diagnosis is uncertain or unavailable.

**Key Points:**

- A CT-based pancreatic radiomics integrated scoring model for PNET (PNET-PRISM) helps when endoscopic ultrasound-guided fine-needle aspiration (EUS-FNA) fails.
- In 407 patients, PRISMPNET-PRISM achieved a high area under the curve (AUC) and 100% external sensitivity for triage.
- The multidimensional deep learning radiomics (M-DLR) score stratified progression-free survival (hazard ratio (HR) ≈ 2.05) and rescued nondiagnostic biopsies.

**Graphical Abstract:**

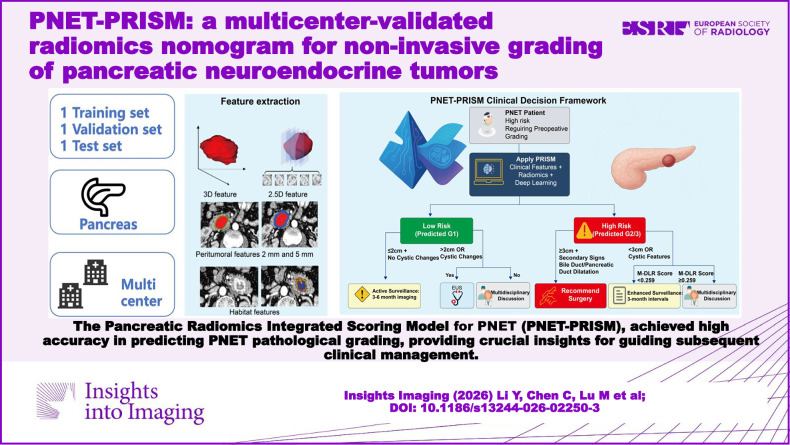

## Introduction

Pancreatic neuroendocrine tumors (PNETs) are a growing clinical challenge, representing about 10% of pancreatic malignancies with a dramatically increasing incidence [1, 2]. The 2019 WHO grading system is a critical determinant of patient outcomes and treatment pathways; 5-year survival rates plummet from 77.3% for grade 1 (G1) to 20.0% for grade 3 (G3) tumors, and guidelines dictate a stark choice between active surveillance for low-grade tumors and immediate surgery for high-grade disease [3–5]. Accurate preoperative grading is therefore paramount. However, the current standard, endoscopic ultrasound-guided fine-needle aspiration (EUS-FNA), is hampered by significant limitations, including sampling errors, procedure-related morbidity, and inadequate tissue yields in up to 40% of cases [6–8]. Consequently, an urgent clinical need exists for a reliable, noninvasive grading alternative.

Radiomics has emerged as a promising noninvasive solution, yet prior studies on PNET grading have suffered from critical methodological flaws that limit their clinical utility. These efforts were typically constrained by single-center designs, small cohorts, and a reliance on simplistic features that failed to capture the complex intratumoral and peritumoral heterogeneity essential for biological characterization [9–11]. While recent advances in artificial intelligence offer powerful tools like automated segmentation and habitat analysis to overcome these limitations, their clinical translation hinges on a crucial step that has been largely unaddressed: rigorous, multicenter validation to ensure model robustness and generalizability across varied imaging protocols and patient populations [12, 13].

**Fig. 1:**
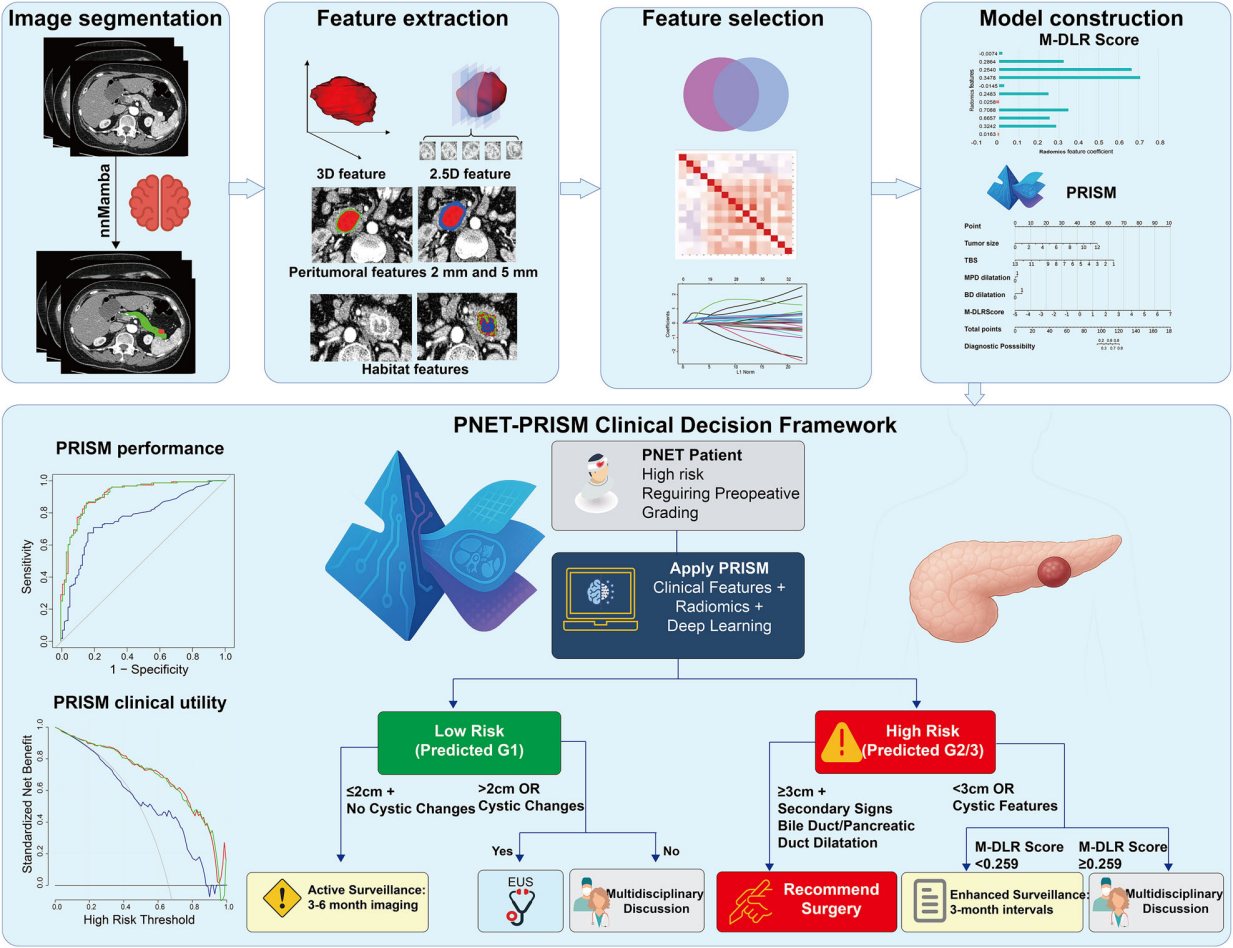
Study design and workflow overview. Complete research framework showing the multicenter study design, from patient recruitment through model development and validation, including the integration of multidimensional radiomics features and clinical variables for PNET grading prediction

To address these critical knowledge and validation gaps, we developed a comprehensive, multidimensional radiomics approach (Fig. 1). This framework integrates state-of-the-art automated segmentation with features extracted from the intratumoral, peritumoral, and habitat domains, as well as deep learning semantic features, to create a holistic tumor profile. The purpose of this multicenter study was therefore to develop and rigorously validate a radiomics-based nomogram for the accurate preoperative prediction of PNET histologic grade, with a particular focus on demonstrating its clinical utility as a powerful complement to invasive tissue sampling procedures.

## Materials and methods

### Patient selection

**Fig. 2:**
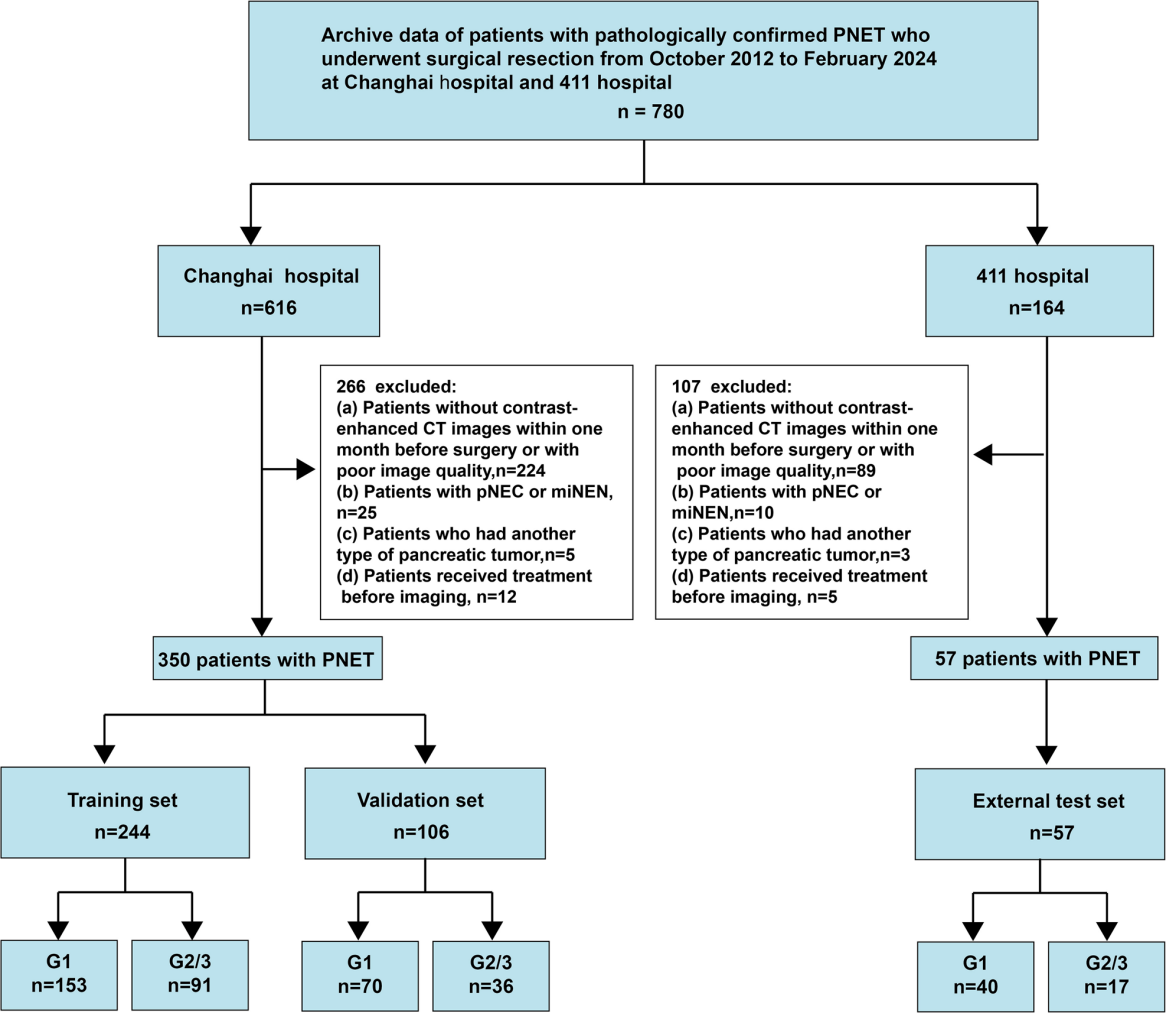
Patient enrollment flowchart. Detailed selection process showing inclusion and exclusion criteria for the multicenter cohort, with temporal division into training (*n* = 244), validation (*n* = 106), and external test sets (*n* = 57)

This study was prepared in accordance with the TRIPOD statement [14] for prediction model development and validation. Reporting of the radiomics workflow [15] and the AI-related methodology was additionally aligned with the CLEAR checklist [16] and the CLAIM (2024 update) checklist [17]. The research received clearance from the Ethics Committee of Changhai Hospital and adhered to stringent ethical standards (Approval No. Changhai-Y2024-019). All imaging and clinical data were de-identified prior to analysis by removing direct identifiers and replacing study IDs with random codes. Data were stored on access-controlled servers within each participating institution, and only authorized study personnel had access for analysis. We consecutively identified 780 patients with PNETs from two institutions between October 2012 and February 2024 (Fig. 2). Inclusion criteria were: (1) surgical resection with pathologically confirmed PNET, and (2) contrast-enhanced CT performed within 1 month before surgery. Exclusion criteria were: (1) prior treatment (chemotherapy, radiation, or targeted therapy), (2) pancreatic neuroendocrine carcinoma or mixed neuroendocrine-non-neuroendocrine neoplasms, and (3) concurrent pancreatic malignancies. Variables required for model development and evaluation were prespecified a priori. Records with missing outcome labels or missing predictor labels were excluded from the analysis. Following TRIPOD guidelines, the Changhai Hospital cohort was temporally divided into training (April 2014–October 2021, *n* = 244) and validation sets (November 2021–January 2024, *n* = 106). The Shanghai 411 Hospital cohort served as an independent external test set (May 2019–December 2021, *n* = 57). We confirmed that none of the patients included in this study overlap with those in our previously published cohorts.

### Sample size calculation

The sample size was estimated using the pmsampsize R package per Riley et al’s guidelines [18]. Based on an expected C-statistic of 0.85, an outcome prevalence (G2/G3 tumors) of 59%, and 5 predictors in the final nomogram—M-DLR Score, tumor diameter, tumor burden score, BD dilatation, and MPD dilatation—the minimum sample required was 198 patients (117 events). To enhance model robustness and generalizability, we targeted a higher events-per-variable (EPV) ratio than the minimum requirement. The final cohort included 407 patients with 240 G2/G3 events, yielding an EPV of 48, substantially exceeding recommended thresholds. A post hoc power analysis confirmed > 95% power for model validation.

### Endpoints

The primary endpoint was the histopathologic grade of PNETs, determined by the Ki-67 proliferation index according to the 2019 WHO classification criteria: G1 (Ki-67 ≤ 3%), G2 (Ki-67 3–20%), and G3 (Ki-67 > 20%). The secondary endpoint was progression-free survival (PFS), defined as the time from surgical resection to the first documented evidence of disease progression, death from any cause, or the last follow-up examination, whichever occurred first.

A rigorous process was implemented to assess PFS objectively and without bias. First, disease progression was strictly defined using radiographic criteria (Response Evaluation Criteria in Solid Tumors, version 1.1), encompassing: (1) local tumor recurrence at the surgical site, (2) development of new distant metastases, or (3) radiographic progression of pre-existing metastatic lesions. Second, all imaging evaluations were conducted by two independent radiologists who were blinded to: (1) PNET-PRISM model predictions, (2) original pathological grade, and (3) clinical treatment decisions. Only anonymized imaging data with unique study IDs were provided to the readers. The inter-reader agreement was high, with a third senior radiologist serving as an arbiter for the few discordant cases (*n* = 8, representing 6.2% of all progression events). Furthermore, although patients underwent clinical and laboratory assessments, including tumor markers (e.g., chromogranin A) during follow-up, a rise in marker levels alone did not constitute progression; instead, it triggered earlier or more detailed radiographic evaluation to confirm progression objectively. This surveillance was carried out according to standardized institutional protocols, with dynamic contrast-enhanced CT or MRI performed every 3–6 months for the initial 2–3 years post-surgery, transitioning to annual examinations until the final follow-up date of July 17, 2024.

### CT image acquisition and analysis

All CT examinations were performed using 256–640 slice scanners (Brilliance iCT, Philips; Aquilion ONE, Canon) with standardized protocols: 120 kV, 150 mAs, 0.5-s rotation time. Non-ionic contrast (350 mg I/mL, 1.5 mL/kg) was administered at 3.5 mL/s through antecubital venous access, followed by 30 mL saline flush. Arterial and portal venous phase images were acquired using automated bolus tracking. A detailed description is available in Supplementary Method 1.

Images were independently reviewed by an abdominal radiologist with > 10 years’ experience, blinded to histopathologic results. Assessed parameters included tumor location, maximum diameter, main pancreatic duct dilatation (> 3 mm), bile duct dilatation (> 10 mm), cystic components, and tumor burden score (TBS, which was defined according to the previously proposed calculation: [TBS² = (maximum tumor diameter)² + (number of tumors)²]).

### Radiomics workflow and model development

Radiomics features were standardized using z-score normalization (mean = 0, SD = 1) based on the training set only. The same scaling parameters were subsequently applied to the validation and external test sets to prevent information leakage. The radiomics analysis comprised a sequential four-part workflow: (1) image segmentation and correction, (2) multidimensional feature extraction, (3) hierarchical feature selection, and (4) predictive model construction.

1. **Image segmentation and correction:** The workflow began with the selection of arterial and portal venous phase images from preoperative contrast-enhanced CT scans. Pancreatic tumors were then segmented using a proprietary automated tool, nnMamba (V1.0/2024SR1384229), which is built upon the nnUNet framework. The automated pipeline demonstrated strong baseline performance, achieving a mean Dice similarity coefficient of 0.88 on an independent test set prior to any manual edits. Following automated segmentation, all segmentations were meticulously reviewed on a slice-by-slice basis by a senior radiologist with over 20 years of experience in pancreatic imaging, using ITK-SNAP software. Manual corrections were performed where necessary to ensure high fidelity, with a target Dice similarity coefficient of ≥ 0.80. A detailed description of the segmentation protocol is available in Supplementary Method 2.
2. **Multidimensional feature extraction:** Following segmentation, a comprehensive, multidimensional feature set was extracted from the delineated tumor volumes. Prior to feature extraction, voxel intensities within each ROI were discretized using a fixed bin width to ensure comparability across scans. The discretization setting (25HU) was kept identical for all cases and all centers, and the same configuration was used for both training and test cohorts. This process yielded five distinct categories of features:

1. **Quantitative morphologic features:** Fundamental measurements including minimum, maximum, and average CT values, as well as tumor volumes and maximum cross-sectional diameters and areas.
2. **2.5D deep learning features:** A hybrid approach that extracted features with 3D contextual information from 2D image slices. This approach analyzes 2D image slices while incorporating contextual information from adjacent slices, offering a balance between 2D computational efficiency and 3D spatial awareness.
3. **Intratumoral and peritumoral radiomic features:** An extensive set of radiomic features characterizing tumor texture and intensity, extracted from both within the tumor and from the surrounding 2-mm and 5-mm peritumoral regions.
4. **Habitat features:** The tumor volume was partitioned into distinct subregions (“habitats”) based on voxel-wise entropy and intensity metrics, from which habitat-specific features were then extracted.
5. **3D radiomic features:** A full set of 3D features was extracted to quantify the tumor’s overall geometric morphology, intensity distribution, and three-dimensional textural patterns.

Detailed methodologies for all feature extraction processes are provided in Supplementary Method 3.

3. **Hierarchical feature selection:** To identify the most informative predictors from the high-dimensional data, a four-step hierarchical feature selection strategy was implemented:

1. **Univariate filtering:** A Mann–Whitney *U*-test was first applied to all extracted features, with only those demonstrating a significant association with tumor grade (*p* < 0.05) being retained.
2. **Redundancy reduction:** To minimize multicollinearity, Spearman correlation analysis was performed. For any pair of features with a correlation coefficient greater than 0.9, one feature was removed.
3. **Recursive elimination:** A greedy recursive elimination algorithm was then used to further refine the feature set by iteratively removing the most redundant feature at each step.
4. **Final feature selection:** Finally, a LASSO (Least Absolute Shrinkage and Selection Operator) logistic regression model with 10-fold cross-validation was employed to select the optimal feature subset used in the final model.

4. **Predictive model construction:** To assess the value of the radiomics signature, two distinct predictive models were developed and compared:

**The clinical model:** Constructed using only significant conventional clinical and radiological characteristics.

**The pancreatic radiomics integrated scoring model (PNET-PRISM):** An integrated model constructed by combining the significant clinical predictors with the final radiomics signature, which was termed the **multidimensional deep learning radiomics score (M-DLR score)**.

### Statistical analysis

Continuous variables were expressed as mean ± standard deviation or median (interquartile range) based on distribution normality. Categorical variables were reported as frequencies and percentages. Group comparisons used *t*-tests, Mann–Whitney *U*-tests, or chi-square tests as appropriate. Model performance was evaluated using receiver-operating characteristic curves, area under the curve (AUC), sensitivity, specificity, positive predictive value, and negative predictive value. Calibration was assessed using Hosmer–Lemeshow tests and calibration curves. Clinical utility was evaluated using decision curve analysis. Model comparisons employed DeLong tests. Net reclassification improvement (NRI) quantified model enhancement. Survival analysis used Kaplan–Meier curves and log-rank tests. Statistical significance was set at *p* < 0.05. All analyses were performed using R software (version 4.3.1).

## Results

### Patient characteristics

The final study cohort included 407 patients with pathologically confirmed PNETs, divided into training (*n* = 244), validation (*n* = 106), and external test (*n* = 57) sets. Overall, the cohort comprised 167 patients with grade 1 (G1) tumors and 240 with grade 2/3 (G2/G3) tumors.

Significant differences were observed between the low- and high-grade groups. In the training and validation cohorts, patients with G2/G3 tumors had significantly larger tumor size, higher tumor burden scores (TBS), and more advanced T, N, and M stages (all *p* < 0.05). In the external test set, these differences were significant for the N category (*p* = 0.040) and the presence of main pancreatic duct dilation (*p* = 0.018). In contrast, other baseline characteristics like patient age and tumor location were comparable between the groups across all datasets. A comprehensive summary of patient and tumor characteristics is provided in Table 1.

**Table 1:** Comparison of patients and tumor characteristics in training, validation and test sets

Characteristics	Training set			Validation set			Test set		
	G1 (*n* = 91)	G2/3 (*n* = 153)	*p*-value	G1 (*n* = 36)	G2/3 (*n* = 70)	*p*-value	G1 (*n* = 40)	G2/3 (*n* = 17)	*p*-value
Age, years	55.60 ± 11.14	52.64 ± 12.81	0.068	53.33 ± 9.23	52.94 ± 12.69	0.870	53.38 ± 11.35	53.00 ± 10.22	0.907
Sex, *n* (%)			0.331			0.131			0.469
Female	54 (59.34%)	81 (52.94%)		21 (58.33)	30 (42.86)		23 (57.50)	8 (47.06)	
Male	37 (40.66%)	72 (47.06%)		15 (41.67)	40 (57.14)		17 (42.50)	9 (52.94)	
Location, *n* (%)			0.086			0.734			0.401
Head and neck	36 (39.56%)	79 (51.63%)		18 (50.00)	30 (42.86)		21 (52.50)	7 (41.18)	
Body and tail	55 (60.44%)	72 (47.06%)		17 (47.22)	36 (51.43)		19 (47.50)	9 (52.94)	
All	0 (0.00%)	2 (1.31%)		1 (2.78)	4 (5.71)		0 (0.00)	1 (5.88)	
Size, cm	2.20 ± 1.50	3.83 ± 2.50	< 0.001	2.26 ± 1.25	4.17 ± 2.89	< 0.001	2.85 ± 2.07	3.58 ± 2.58	0.264
Number, *n* (%)			0.053			0.942			1.000
1	86 (94.51)	152 (99.35)		32 (88.89)	64 (91.43)		38 (95.00)	17 (100.00)	
≥ 2	5 (5.49)	1 (0.65)		4 (11.11)	6 (8.57)		1 (5.00)	0 (0.00)	
TBS	2.54 ± 1.41	4.01 ± 2.42	< 0.001	2.63 ± 1.18	4.38 ± 2.81	< 0.001	3.13 ± 1.98	3.78 ± 2.49	0.299
T category, *n* (%)			< 0.001			0.001			0.138
T1	48 (52.75)	33 (21.57)		18 (50.00)	11 (15.71)		16 (40.00)	3 (17.65)	
T2	34 (37.36)	58 (37.91)		13 (36.11)	33 (47.14)		14 (35.00)	6 (35.29)	
T3	9 (9.89)	53 (34.64)		5 (13.89)	21 (30.00)		10 (25.00)	7 (41.18)	
T4	0 (0.00)	9 (5.88)		0 (0.00)	5 (7.14)		0 (0.00)	1 (5.88)	
N category, *n* (%)			< 0.001			0.005			0.040
N0	85 (93.41)	108 (70.59)		33 (91.67)	44 (62.86)		39 (97.50)	13 (76.47)	
N1-2	6 (6.59)	45 (29.41)		3 (8.33)	26 (37.14)		1 (2.50)	4 (23.53)	
M category, *n* (%)			0.008			0.049			0.085
M0	88 (96.70)	132 (86.27)		34 (94.44)	56 (80.00)		40 (100.00)	15 (88.24)	
M1-2	3 (3.30)	21 (13.73)		2 (5.56)	14 (20.00)		0 (0.00)	2 (11.76)	
Cystic, *n* (%)			0.592			0.543			0.432
No	85 (93.41)	140 (91.50)		32 (88.89)	66 (94.29)		36 (90.00)	17 (100.00)	
Yes	6 (6.59)	13 (8.50)		4 (11.11)	4 (5.71)		4 (10.00)	0 (0.00)	
MPD dilatation, *n* (%)			0.029			0.200			0.018
No	72 (79.12)	101 (66.01)		28 (77.78)	46 (65.71)		36 (90.00)	10 (58.82)	
Yes	19 (20.88)	52 (33.99)		8 (22.22)	24 (34.29)		4 (10.00)	7 (41.18)	
BD dilatation, *n* (%)			0.008			0.246			0.209
No	88 (96.70)	132 (86.27)		36 (100.00)	65 (92.86)		39 (97.50)	15 (88.24)	
Yes	3 (3.30)	21 (13.73)		0 (0.00)	5 (7.14)		1 (2.50)	2 (11.76)	

*TBS* tumor burden score, *BD* bile duct, *MPD* dilatation of the main pancreatic duct

### Construction and validation of the M-DLR score

**Fig. 3:**
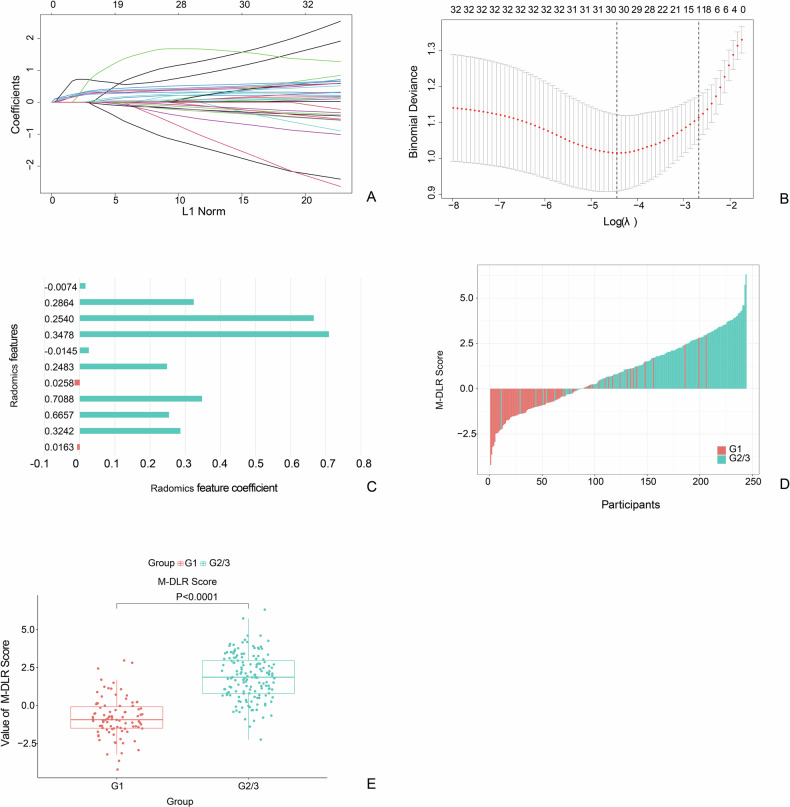
Development and validation of the M-DLR score in the training set. **A** Selection of the tuning parameter (λ) in the LASSO model via 10-fold cross-validation based on the minimum criteria. Binomial deviances from the LASSO regression cross-validation procedure were plotted as a function of log(λ). The *y*-axis indicates binomial deviances; the lower *x*-axis indicates log(λ). Numbers along the upper *x*-axis represent the average number of predictors. Red dots indicate average deviance values for each model with a given λ, and vertical bars show the upper and lower deviance values. Vertical black lines define optimal λ values where the model provides the best fit. The optimal λ value of 0.0069 with log(λ) = −2.673 was selected. **B** LASSO coefficient profiles of the 11 selected features. The dotted vertical line corresponds to the optimal λ value selected in **A**. **C** Feature coefficient trajectories demonstrating the impact of regularization parameter variation on selected radiomics features. **D** Waterfall plot displaying M-DLR Score distribution across patients, with red bars representing G1 tumors and blue bars representing G2/3 tumors. **E** Scatter plot comparison of M-DLR Score values between G1 and G2/3 groups. Each dot represents an individual patient’s M-DLR score. Horizontal lines represent group means. Statistical significance: *p* < 0.0001. M-DLR, multidimensional deep learning radiomics; LASSO, least absolute shrinkage and selection operator

From an initial set of over 13,000 multidimensional features, a hierarchical screening framework selected an optimal subset of 11 features to construct the multidimensional deep learning radiomics score (M-DLR Score) (Fig. 3C).

The resulting M-DLR Score demonstrated a highly significant difference between the G1 and G2/G3 tumor groups across all datasets (*p* < 0.0001) (Fig. 3D). The median scores consistently distinguished the two groups in the training set (G1: −0.94 vs. G2/3: 1.85), validation set (G1: −0.31 vs. G2/3: 2.38), and test set (G1: −0.19 vs. G2/3: 1.89).

**Fig. 4:**
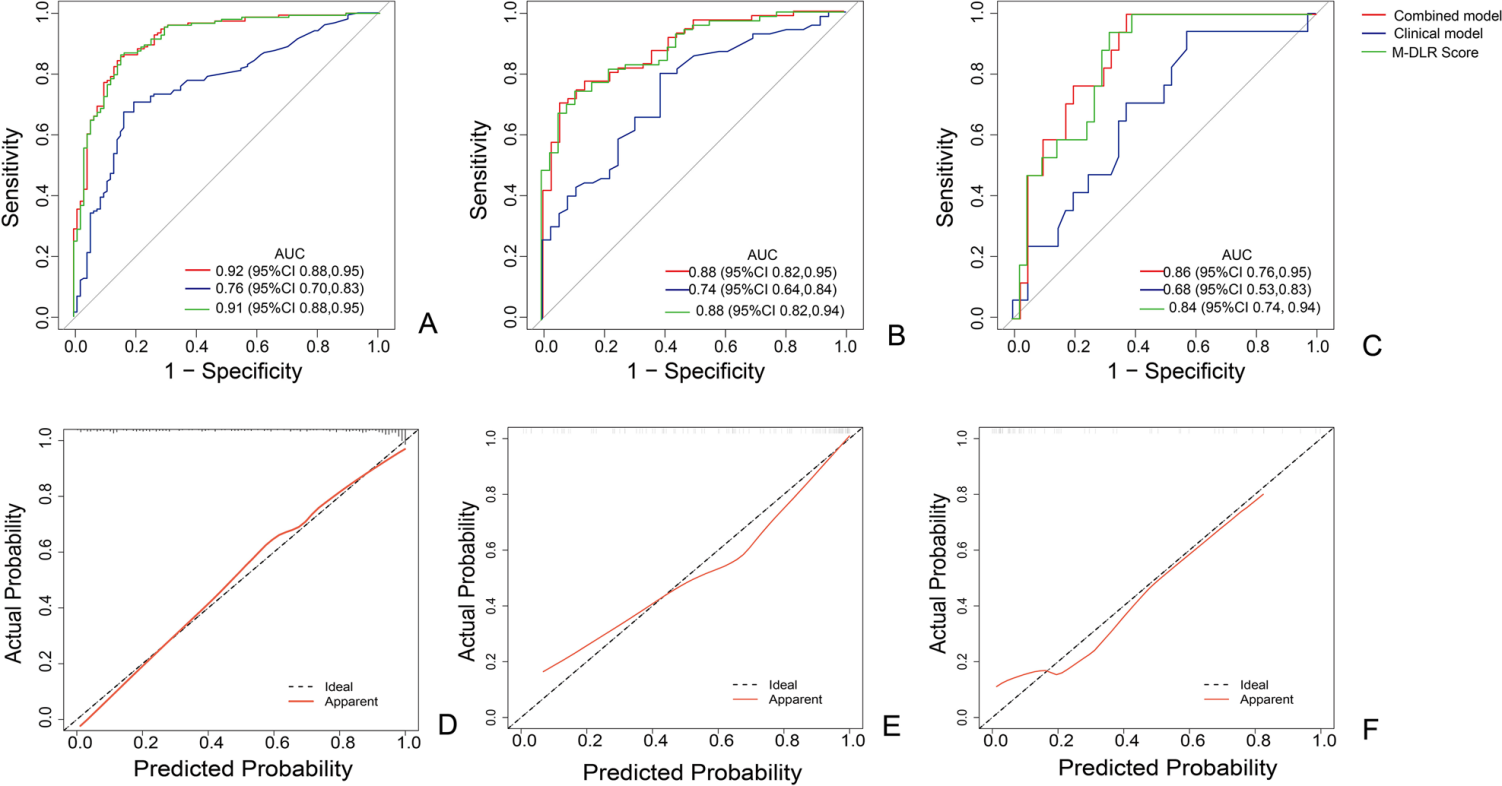
Model performance evaluation and calibration analysis. **A**–**C** Receiver-operating characteristic (ROC) curves comparing clinical model, M-DLR score alone, and pancreatic radiomics integrated scoring model for PNET (PNET-PRISM) across training, validation, and external test sets, with corresponding area under the curve (AUC) values and 95% confidence intervals. **D**–**F** Calibration curves for PNET-PRISM across training, validation, and external test sets, demonstrating agreement between predicted probabilities and observed outcomes (Hosmer–Lemeshow test: *p* > 0.05 for all datasets). M-DLR, multidimensional deep learning radiomics; PNET, Pancreatic neuroendocrine tumor

The M-DLR Score alone yielded robust diagnostic performance for grading, with AUCs of 0.91 (95% CI, 0.88–0.94) in the training set, 0.88 (95% CI, 0.82–0.93) in the validation set, and 0.84 (95% CI, 0.75–0.92) in the test set. Detailed performance metrics are available in Table 2 and Fig. 4.

**Table 2:** The performance of the clinical model, M-DLR score and PRISMs

Performance	Training set			Validation set			Test set		
	Clinical model	M-DLR Score	PRISM	Clinical model	M-DLR Score	PRISM	Clinical model	M-DLR Score	PRISM
AUC (95% CI)	0.77 (0.71–0.83)	0.91 (0.88–0.95)	0.92 (0.88–0.95)	0.80 (0.71–0.88)	0.88 (0.81–0.94)	0.89 (0.82–0.94)	0.71 (0.56–0.85)	0.84 (0.73–0.93)	0.87 (0.76–0.94)
Sensitivity	0.70 (107/153)	0.86 (132/153)	0.86 (132/153)	0.54 (37/69)	0.74 (52/70)	0.77 (53/69)	0.76 (13/17)	0.94 (16/17)	1.0 (17/17)
Specificity	0.79 (72/91)	0.85 (77/91)	0.86 (78/91)	0.95 (35/37)	0.89 (32/36)	0.86 (32/37)	0.60 (24/40)	0.68 (27/40)	0.68 (27/40)
Accuracy	0.73 (179/244)	0.86 (209/244)	0.86 (210/244)	0.68 (72/106)	0.79 (84/106)	0.80 (85/106)	0.65 (37/57)	0.75 (43/57)	0.77 (44/57)
PPV	0.85 (107/126)	0.90 (132/146)	0.91 (132/145)	0.95 (37/39)	0.93 (52/56)	0.91 (53/58)	0.45 (13/29)	0.55 (16/29)	0.57 (17/30)
NPV	0.61 (72/118)	0.79 (77/98)	0.79 (78/99)	0.52 (35/67)	0.64 (32/50)	0.67 (32/48)	0.86 (24/28)	0.96 (27/28)	1.00 (27/27)

*PRISM* pancreatic radiomics integrated scoring model, *CI* confidence interval, *AUC* area under the curve, *PPV* positive predictive value, *NPV* negative predictive value, *M-DLR* multidimensional deep learning radiomics

### Association of M-DLR score with progression-free survival (PFS)

Using an optimal cutoff value of 0.259, the M-DLR Score was used to stratify patients into low-risk and high-risk groups. Kaplan–Meier analysis revealed that patients in the low-risk group had significantly longer progression-free survival (PFS) compared to the high-risk group across the training (*p* = 0.0066), validation (*p* < 0.0001), and external test sets (*p* = 0.0197). Overall, the log-rank test confirmed that the M-DLR Score was significantly associated with PFS (HR, 2.050, 95% CI, 1.484–2.833, *p* < 0.001), indicating its prognostic value (Supplementary Fig. 1).

### Performance of predictive models

Univariate analysis identified that tumor size, TBS, main pancreatic duct dilation, common bile duct dilation, and the M-DLR Score were all significant predictors of PNET grade (all *p* < 0.05) (Table 3).

**Table 3:** The univariable logistic regression analysis between G grades and all variables

Characteristic	B	SE	CI	*p*-value
BD dilatation	1.54	0.63	4.67 (1.55–20.17)	0.015
MPD dilatation	0.67	0.31	1.95 (1.08–3.64)	0.031
TBS	0.48	0.10	1.61 (1.33–2.01)	< 0.001
Tumor size	0.49	0.10	1.63 (1.36–2.03)	< 0.001
Cystic	0.27	0.51	1.32 (0.50–3.863)	0.593
Sex	0.26	0.27	1.30 (0.77–2.20)	0.331
Age	−0.02	0.01	0.98 (0.96–1.00)	0.069
M-DLR score	1.37	0.17	3.94 (2.89–5.71)	< 0.001

*SE* standard error, *CI* confidence interval, *TBS* tumor burden score, *BD* bile duct, *MPD* dilatation of the main pancreatic duct, *M-DLR score* multidimensional imaging omics deep learning score

Based on these findings, two predictive models were developed. The clinical model, using only clinical and radiological variables, demonstrated moderate discriminative power, with AUCs of 0.77, 0.80, and 0.71 in the training, validation, and test sets, respectively (Table 4).

**Table 4:** Multivariate logistic regression analysis of clinical model

Characteristic	B	SE	OR (95% CI)	*p*-value
(Intercept)	0.05	0.754	1.051 (0.293–5.244)	0.947
Tumor size	2.985	1.668	19.77 (1.811–910.5)	0.073
TBS	−2.68	1.764	0.068 (0.001–0.870)	0.129
BD dilatation	0.499	0.342	1.647 (0.849–3.262)	0.144
MPD dilatation	1.185	0.668	3.270 (0.993–14.84)	0.076

Clinical model = 0.05 + 2.98 × tumor size − 2.68 × TBS + 0.499 × BD dilatation + 1.185 × MPD dilatation

*SE* standard error, *OR* odds ratio, *CI* confidence interval, *TBS* tumor burden score, *BD* bile duct, *MPD* dilatation of the main pancreatic duct

**Fig. 5:**
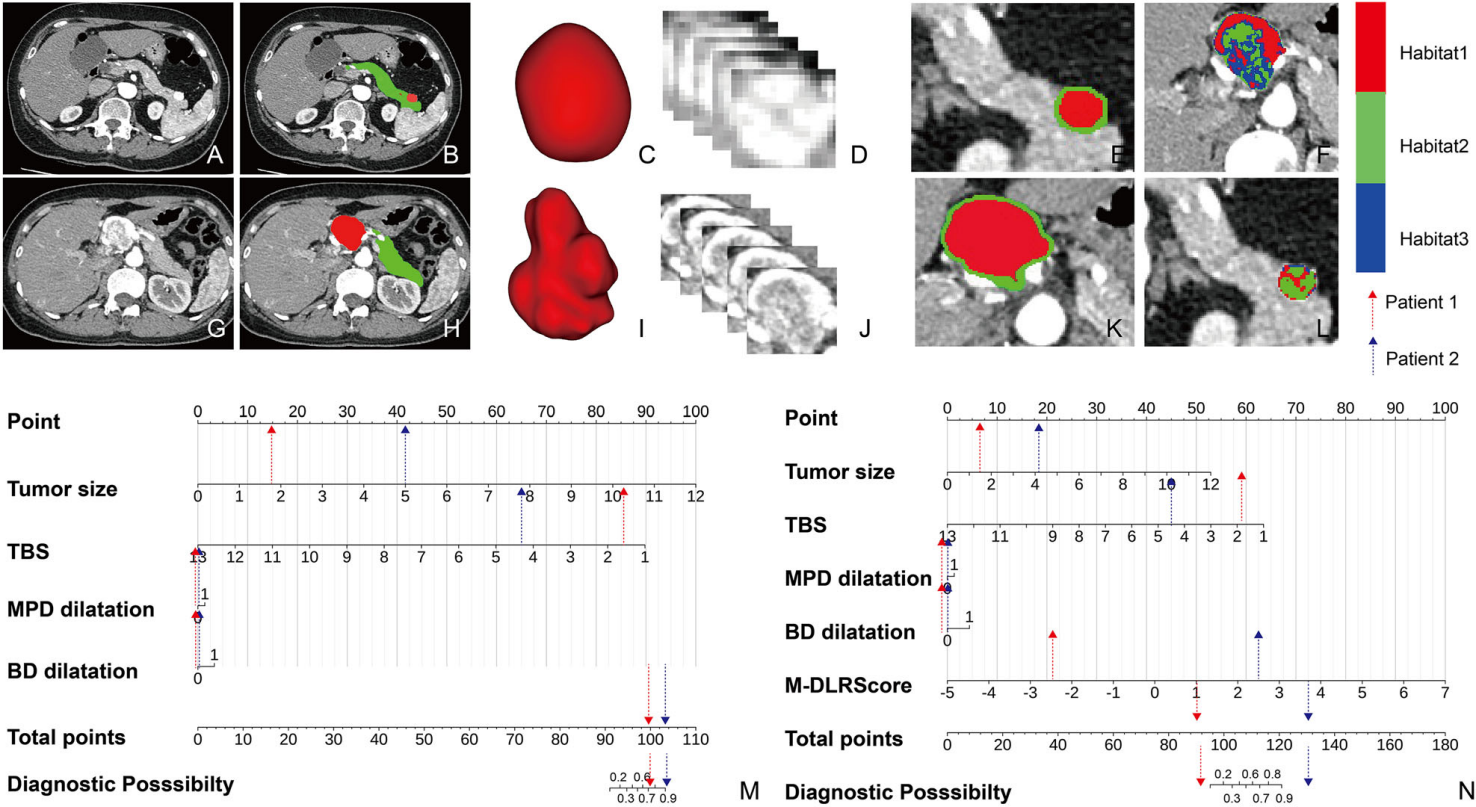
Representative clinical cases demonstrating pancreatic radiomics integrated scoring model for PNET (PNET-PRISM) utility. Top panel: G1 PNET correctly classified by PNET-PRISM. A 72-year-old woman with a 1.6-cm pancreatic body-tail tumor (T1N0M0). Despite clinical features suggesting higher grade (tumor size, TBS 1.87), the M-DLR Score of −2.48 enabled correct G1 classification, with favorable outcomes at 19.17-month follow-up. **A** Arterial phase CT image; **B** automated segmentation (red: tumor, green: pancreatic parenchyma); **C** 3D tumor rendering; **D** 2.5D feature extraction schematic showing 5-layer analysis; **E** tumor region with 2-mm peritumoral zone (green); **F** habitat analysis displaying 3-cluster segmentation. Bottom panel: G2 PNET correctly upgraded by PNET-PRISM. A 50-year-old woman with a 4.3-cm pancreatic head tumor (T2N0M0). While clinical features alone suggested G1, incorporation of M-DLR Score (2.660) correctly identified G2/3 grade and high-risk status, validated by subsequent liver metastasis development within 1 month post-surgery. **G**–**L** Corresponding imaging analyses as described above. Nomograms: Visual representation of **M** clinical model incorporating conventional radiological and clinical variables, and **N** PNET-PRISM integrating clinical predictors with M-DLR Score for improved PNET grade prediction accuracy

The PNET-PRISM, which integrated the M-DLR Score with the clinical predictors, showed markedly superior performance (Table 5). It achieved AUCs of 0.92 (95% CI, 0.88–0.95), 0.89 (95% CI, 0.82–0.94), and 0.87 (95% CI, 0.76–0.94) in the training, validation, and test sets, respectively (Table 2, Fig. 4). Calibration curves indicated good model fit across all datasets (Hosmer–Lemeshow test, *p* > 0.15 for all) (Fig. 4). Representative cases illustrating the model’s utility are shown in Fig. 5.

**Table 5:** Multivariate logistic regression analysis of the PRISM

Characteristic	B	SE	OR (95% CI)	*p*-value
(Intercept)	0.428	0.670	1.533 (0.488–6.609)	0.523
M-DLR score	1.417	0.196	4.123 (2.890–6.247)	< 0.001
Tumor size	0.750	1.412	2.115 (0.284–59.91)	0.595
TBS	−0.900	1.473	0.406 (0.012–3.256)	0.541
BD dilatation	0.754	0.930	2.125 (0.399–16.32)	0.418
MPD dilatation	0.235	0.439	1.264 (0.535–3.013)	0.593

PRISM = 0.428 + 1.417 × M-DLR Score + 0.75 × tumor size − 0.900 × TBS + 0.754 × BD dilatation + 0.235 × main pancreatic duct dilation

*PRISM* pancreatic radiomics integrated scoring model, *SE* standard error, *OR* odds ratio, *CI* confidence interval, *M-DLR score* multidimensional imaging omics deep learning score, *TBS* tumor burden score, *BD* bile duct, *MPD* dilatation of the main pancreatic duct

### Model comparisons and clinical utility

Model comparisons confirmed that PNET-PRISM significantly outperformed the Clinical Model in all datasets (DeLong test, *p* < 0.05). While its performance was comparable to the M-DLR Score alone, Net Reclassification Improvement (NRI) analysis demonstrated its superior accuracy, with significant gains over the Clinical Model in the training (NRI = 0.406, *p* < 0.001), validation (NRI = 0.127, *p* = 0.030), and test sets (NRI = 0.318, *p* = 0.070) (Supplementary Fig. 2).

**Fig. 6:**
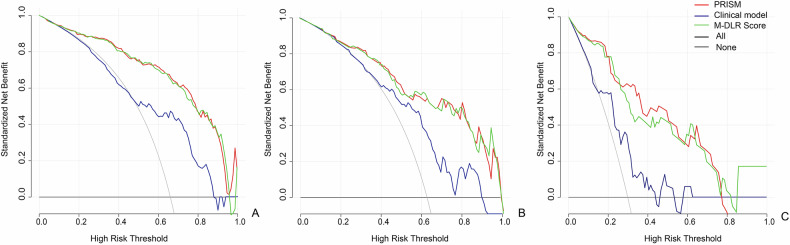
Decision curve analysis for clinical utility assessment. Net benefit curves for M-DLR Score, pancreatic radiomics integrated scoring model for PNET (PNET-PRISM), and clinical model across **A** training, **B** validation, and **C** external test sets. The *x*-axis represents the threshold probability for treatment decisions; the *y*-axis shows net benefit. Gray line indicates “treat all as G2/3” strategy; black line indicates “treat all as G1” strategy. PNET-PRISM demonstrates superior clinical utility with greater net benefit across most threshold probabilities, indicating improved patient outcomes compared to alternative strategies. M-DLR, multidimensional deep learning radiomics; PNET, Pancreatic neuroendocrine tumor

In a head-to-head comparison with EUS-FNA (*n* = 40), PNET-PRISM demonstrated significant clinical value. Among the 25 patients for whom EUS-FNA was nondiagnostic—primarily due to insufficient cellularity (12/25, 48%), extensive necrosis (7/25, 28%), or sampling error (6/25, 24%)—PNET-PRISM provided accurate grading in 13 cases (52%). Conversely, in cases with successful biopsies, its accuracy was comparable to EUS-FNA (*p* > 0.05). Finally, decision curve analysis confirmed that using PNET-PRISM to guide clinical decisions provides a superior net benefit across a wide range of threshold probabilities in all three cohorts (Fig. 6).

### Analysis of misclassified cases

To better understand the limitations of PNET-PRISM, we conducted a detailed error analysis on the 73 (17.9%) misclassified cases among 407 patients, with error rates of 14.8%, 20.8%, and 26.3% in the training, validation, and external test sets, respectively. False positives (G1 tumors misclassified as G2/G3; *n* = 33) were primarily associated with larger tumor size (3.45 ± 1.88 cm vs. 2.10 ± 1.44 cm for correct G1; *p* < 0.001) and cystic changes, indicating that large G1 tumors with atypical imaging features are prone to over-grading. Conversely, false negatives (G2/G3 tumors misclassified as G1; *n* = 40) were linked to smaller tumors (2.33 ± 2.43 cm vs. 4.22 ± 2.55 cm for correct G2/G3; *p* < 0.001) lacking secondary signs of aggression, highlighting a diagnostic challenge for early-stage malignancies with indolent phenotypes. Based on these failure modes, we propose a risk-stratified clinical decision framework that integrates PNET-PRISM predictions with tumor size and cystic features to guide management, such as surveillance for low-risk predicted G1 tumors (≤ 2 cm) versus EUS-FNA or multidisciplinary discussion for higher-risk or equivocal cases (Supplementary Figs. 3–5, Supplementary Table 2).

## Discussion

The preoperative grading of pancreatic neuroendocrine tumors (PNETs) represents a critical clinical fork in the road, dictating a profound choice between invasive surgery and active surveillance. Yet, the current clinical standard, endoscopic ultrasound-guided fine-needle aspiration (EUS-FNA), often leads to a dead end; it is invasive, carries procedural risks, and fails to provide a definitive diagnosis in up to 40% of cases due to inadequate tissue sampling. This impasse has created an urgent, unmet need for a reliable, noninvasive tool to guide treatment decisions. While prior CT radiomics studies have attempted to fill this gap, they have been hampered by single-center designs, reliance on simplistic features, and a failure to account for the complex tumor microenvironment, limiting their clinical translation and leaving the core problem unsolved.

To break this stalemate, we developed and rigorously validated PNET-PRISM, a multidimensional radiomics nomogram designed to function as a “virtual biopsy.” Our approach addresses the fundamental flaws of previous models through four key innovations: (1) fully automated, deep learning-based segmentation to ensure reproducibility; (2) systematic analysis of the peritumoral microenvironment, capturing tumor-host interactions invisible to the naked eye; (3) intratumoral habitat analysis to quantify regional heterogeneity, mirroring pathological variation; and (4) the integration of advanced deep learning features to capture complex semantic patterns. This multicenter study, with its robust external validation, was specifically designed to prove that such a comprehensive approach could yield a clinically translatable tool that is both generalizable and reliable across different institutions.

Our results demonstrate that PNET-PRISM not only achieves exceptional diagnostic accuracy—with AUCs of 0.92, 0.89, and 0.87 across the training, validation, and external test cohorts, respectively—but also provides deeper biological and prognostic insights. The model’s performance is not a “black box”; its predictive power is driven by features with strong biological plausibility. For instance, the high importance of features from the 2 mm peritumoral region likely reflects underlying biological processes of high-grade tumors, such as neo-angiogenesis and inflammatory infiltration at the tumor-stroma interface. Similarly, habitat features identifying hypovascular, high-entropy regions correspond well with tumor necrosis and hypoxia, hallmarks of aggressive biology.

Crucially, the clinical value of PNET-PRISM extends beyond diagnosis. Its radiomic signature, the M-DLR Score, functions as a powerful independent prognostic biomarker, with higher scores being significantly associated with shorter progression-free survival (HR = 2.050, *p* < 0.001). This dual diagnostic and prognostic capability elevates PNET-PRISM from a simple grading tool to a comprehensive risk-stratification instrument. Furthermore, its ability to correctly grade 52% of cases where EUS-FNA failed to yield a diagnosis—overcoming key limitations such as insufficient cellularity, necrosis, and sampling error—underscores its direct utility in resolving clinical uncertainty. The model’s perfect sensitivity (100%) in the external validation cohort, even at the cost of specificity, reflects a conservative bias that prioritizes patient safety. This is particularly valuable when EUS-FNA is nondiagnostic or contraindicated, making it an ideal tool to confidently identify patients who require aggressive management.

This work offers a new insight with the potential to shift the clinical paradigm for PNET management. We demonstrate that routine, noninvasive CT scans contain a wealth of untapped biological and prognostic information that, when unlocked by a multidimensional AI framework, can reliably guide critical treatment decisions. This moves beyond merely complementing the existing standard; it provides a robust solution precisely where the standard fails.

The analysis of our model’s failure modes further illuminates the complex biology of PNETs, where some low-grade tumors exhibit aggressive imaging features, and some high-grade tumors appear indolent. This understanding allowed us to develop a tangible clinical contribution: a novel, risk-stratified decision framework that integrates PNET-PRISM’s prediction with tumor size and features to guide clinicians on whether to pursue surveillance, EUS-FNA, or immediate surgery. This data-driven framework provides a clear, actionable pathway for personalizing patient care.

This study provides a solid foundation for future research, built upon transparently acknowledged limitations. Its retrospective nature and surgically exclusive cohort mean that prospective validation in non-surgical and surveillance populations is the critical next step. Furthermore, the combination of G2 and G3 tumors, while clinically justified by current resection guidelines, may mask unique biological characteristics of G3 tumors that warrant further investigation.

Future research should build directly upon this foundation by: (1) conducting prospective trials to confirm PNET-PRISM’s clinical utility and cost-effectiveness; (2) developing models specifically to distinguish G3 tumors, potentially by incorporating mitosis-specific features; and (3) integrating radiomics data with other promising biomarkers, such as ctDNA, to create even more powerful multi-modal prediction tools.

Regarding clinical implementation, PNET-PRISM could be deployed either as a standalone cloud-based tool or integrated directly into hospital PACS systems for seamless workflow adoption. To facilitate translation, we have publicly released the prediction models and inference code on GitHub (“Data availability” section). Our team is exploring collaborations for prospective trials and software prototyping, with pilot studies anticipated.

In conclusion, this study presents a rigorously validated, multidimensional radiomics nomogram that effectively addresses a critical unmet need in PNET management. By providing a reliable, noninvasive tool for both grading and prognosis, PNET-PRISM offers a robust foundation for optimizing clinical decision-making, personalizing treatment pathways, and ultimately improving patient outcomes.

## Supplementary information

ELECTRONIC SUPPLEMENTARY MATERIAL

## Data Availability

Data supporting the findings are available from the corresponding authors upon reasonable request.

## Abbreviations

2.5D: Two-and-a-half dimensional (2D slices with 3D context)
AUC: Area under the receiver-operating characteristic curve
EUS-FNA: Endoscopic ultrasound-guided fine-needle aspiration
HR: Hazard ratio
M-DLR Score: Multidimensional deep learning radiomics score (model component)
PFS: Progression-free survival
PNET(s): Pancreatic neuroendocrine tumor(s)
PNET-PRISM: Pancreatic Radiomics Integrated Scoring Model for PNET
TBS: Tumor burden score (TBS² = diameter^2^ + number^2^)

## Code availability

The prediction models and inference code are publicly available at the authors’ GitHub repository (Changhai-AILab/Net-Radiomics).
