# Supplementary material for: PNET-PRISM: a multicenter-validated radiomics nomogram for noninvasive grading of pancreatic neuroendocrine tumors
Source: Insights Imaging. 2026 Mar 24;17:78. doi: 10.1186/s13244-026-02250-3 (PMC13013714; doi:10.1186/s13244-026-02250-3)
Supplement: Supplementary file 1 — ELECTRONIC SUPPLEMENTARY MATERIAL [file 13244_2026_2250_MOESM1_ESM.pdf]

PNET-PRISM: A Multicenter-Validated Radiomics Nomogram for Non-invasive Grading of Pancreatic Neuroendocrine Tumors

ELECTRONIC SUPPLEMENTARY MATERIAL

Content

Supplementary method 1. CT image preparation 2

Supplementary method 2. Image segmentation and correction................2

Supplementary method 3: Features extraction 4

Supplementary method 4: Computational Environment............................6

Supplementary Table 1. Construction of M-DLR Score.............................8

Supplementary Table 2: Clinical and Imaging Characteristics of Misclassified Cases 9

Supplementary Figure 1： Kaplan-Meier survival curves for progression-free survival stratified by M-DLR Score risk groups. 10

Supplementary Figure 2: Net Reclassification Improvement (NRI) analysis demonstrating PNET-PRISM's superior performance over the Clinical Model. 12

Supplementary Figure 3: Representative false positive cases: G1 PNETs misclassified as G2/3 by the PNET-PRISM model..14

Supplementary Figure 4. Representative false negative cases: G2/3 PNETs misclassified as G1 by the PNET-PRISM model................................16

Supplementary Figure 5: Clinical decision-making flowchart for PNET-PRISM (Pancreatic Radiomics Integrated Scoring Model) implementation in PNET management. 18

Supplementary method 1. CT image preparation

All CT examinations were performed using either 640-slice CT scanners (Aquilion ONE, Canon Medical Systems, Japan) or 256-slice CT scanners (Brilliance iCT, Philips Healthcare, the Netherlands). Standardized scanning parameters were applied across both platforms: tube voltage 120 kV, tube current 150 mAs, beam collimation 160 mm × 0.5 mm, reconstruction matrix 350 × 350 pixels, and gantry rotation time 0.5 seconds. The timing for enhanced scanning phases was determined using real-time bolus tracking technology (Care-Bolus). A non-ionic iodinated contrast medium with an iodine concentration of 350 mg I/mL was administered intravenously at a weight-based dose of 1.5 mL/kg body weight through an antecubital venous access via a high-pressure injector at a rate of 3.5 mL/s, immediately followed by a 30 mL normal saline flush at the same injection rate to ensure complete contrast delivery. Following contrast injection, multiphase enhanced scanning was performed, including arterial phase (20-25 seconds post-injection), portal venous phase (60-70 seconds post-injection), and delayed phase (110-130 seconds post-injection). The scanning field of view extended from the diaphragmatic level to the pelvic inlet, ensuring comprehensive coverage of the pancreas and potential metastatic sites for complete tumor assessment.

Supplementary method 2. Image segmentation and correction

1) Image Preprocessing

Image preprocessing is essential for preparing medical imaging data for deep learning models. First, normalization standardizes pixel values to a uniform range, with all pixel intensities scaled to [-1, 1] in this study. This normalization enhances training stability and reduces model sensitivity to variations in imaging equipment and acquisition protocols, ensuring robust generalization across different scanner types and imaging settings. Second, comprehensive data augmentation techniques were implemented to increase training data diversity, prevent overfitting, and improve model generalization capability. The nnMamba framework employs multiple augmentation strategies,

including random rotations, translations, scaling transformations, and image flipping operations. These transformations simulate various spatial variations in clinical images, rendering the model invariant to changes in image orientation and anatomical positioning. Additionally, random cropping and controlled noise injection were incorporated into the augmentation pipeline to replicate real-world clinical imaging conditions, thereby enhancing model robustness and reliability.

2) Image Segmentation Model Training

The nnMamba framework was developed as an enhanced version of the established nnUNet architecture, which builds upon the foundational U-Net model for medical image segmentation. Our nnMamba implementation incorporates advanced depthwise separable convolutions, which decompose standard convolution operations into two distinct computational steps, significantly reducing parameter count and computational overhead while improving both training efficiency and segmentation accuracy. The nnMamba framework demonstrates adaptive capabilities across diverse medical imaging datasets by automatically optimizing hyperparameters according to dataset-specific characteristics. During model training, a composite loss function combining Dice loss with cross-entropy loss was employed to optimize segmentation performance. Training parameters were configured as follows: initial learning rate of 0.0001, total training iterations of 1000 epochs, momentum coefficient of 0.99, and the Adam optimizer for weight updates to facilitate convergence toward optimal model performance.

3) Segmentation Performance Assessment

Following training completion, the nnMamba model underwent comprehensive evaluation using an independent test dataset to assess real-world application performance. The evaluation protocol included detailed visualization of segmentation results, enabling qualitative assessment of prediction quality through direct comparison between model-generated segmentations and ground truth annotations. Quantitative performance metrics,

including Dice similarity coefficient, sensitivity, and specificity, were calculated to provide objective measures of segmentation accuracy and reliability for clinical implementation.

Supplementary method 3: Features extraction

1) Quantitative Morphological Features

CT Value Analysis: Fundamental intensity characteristics were extracted from pixels within the segmentation mask, including minimum, mean, and maximum CT values across 2D, 2.5D, and 3D tumor representations. These Hounsfield unit measurements provide quantitative assessment of tissue density and composition heterogeneity.

Maximum Diameter Measurement: The maximum tumor diameter was determined as the largest measurable distance across the lesion in any cross-sectional plane. Following tumor segmentation, binary image processing was performed to isolate the target region. The algorithm identified the largest inscribed circle (2D) or sphere (3D) within each tumor structure, with geometric boundaries precisely touching the lesion margins. Center coordinates and radial measurements were computed using OpenCV polygon detection functions and cv2.minMaxLoc algorithms to quantify maximum tumor dimensions accurately.

Maximum Cross-Sectional Area: Comprehensive planar sectioning was performed across multiple orientations through the 3D tumor volume, calculating individual cross-sectional areas and identifying the largest planar dimension as the maximum cross-sectional area.

Total Volume Calculation: Following deep learning-based tissue segmentation, non-zero-pixel enumeration was performed across sequential image slices for each patient. Three-dimensional volume computation incorporated pixel spacing and slice thickness parameters using the formula: $= \sum_{i=0}^{k} P_i * S_{xi} * S_{yi} * C$, where K represents the number of sections, P_i denotes total pixels in consecutive target structure layers, S_xi and S_yi indicate pixel

spacing in x and y directions respectively, and C represents scan slice thickness.

2) 2.5D Deep Learning Features

Two sophisticated pre-trained models were employed for 2.5D feature extraction: ResNet18 and the advanced vision transformer DINOv2. Feature extraction was performed on five consecutive slices centered on the largest tumor cross-section (positions -4, -2, 0, +2, +4 relative to the reference slice). ResNet18, pre-trained on ImageNet for natural image classification, extracted 512 features per slice, yielding 2,560 total features (5 slices × 512 features). DINOv2, a self-supervised vision model trained on large-scale natural image datasets, demonstrated superior image representation capabilities by extracting 1,024 features per slice, totaling 5,120 features (5 slices × 1,024 features). The DINOv2 model excels at capturing global contextual patterns and complex spatial relationships, making it particularly suitable for comprehensive lesion characterization and providing robust feature representations for accurate tumor identification.

3) Intratumoral and Peritumoral Radiomic Features

Peritumoral microenvironment analysis was performed using the SimpleITK Python package (v.3.6) [1] to generate expanded regions of interest. The original tumor segmentation mask was radially dilated outward from tumor boundaries at 1mm increments to create 2mm and 5mm peritumoral zones. Eight distinct image transformation filters were applied to both expansion regions: wavelet transform, square transform, square root transform, two-dimensional local binary patterns, Laplacian of Gaussian, logarithmic transform, exponential transform, and gradient transform. This comprehensive filtering approach generated 1,409 dimensional features per region, encompassing first-order statistical measures and higher-order texture characteristics, resulting in 2,818 total peritumoral features. Detailed feature specifications and computational methodologies are available in the official PyRadiomics documentation.

4) Habitat Feature Analysis

Tumor habitat mapping was performed using K-means clustering to identify distinct intra-tumoral subregions based on voxel intensity and entropy values. Euclidean distance metrics incorporating both voxel intensity and entropy characteristics were used to determine sample relationships. Optimal cluster number determination was achieved through Consensus Cluster Plus methodology, testing k values from 2 to 10, with k=3 identified as optimal. Image standardization included resampling to $1 \times 1 \times 1 mm^3$ voxel dimensions and intensity normalization to 0-255 grayscale range to address reconstruction variations across different acquisition protocols. PyRadiomics-based feature extraction was performed independently for each habitat subregion, generating three categories of handcrafted features: 14 geometric shape descriptors, 18 first-order intensity characteristics, and 75 texture complexity measures. This approach yielded 107 features per subregion, totaling 321 habitat-specific features for comprehensive intratumoral heterogeneity quantification.

5) 3D Radiomic Features

Comprehensive three-dimensional radiomic analysis encompassed seven distinct feature categories: morphological characteristics (including 3D tumor diameter, volume, surface area, maximum diameter, and length-width ratio), first-order statistical measures, gray-level co-occurrence matrix (GLCM) features, gray-level dependence matrix (GLDM) features, gray-level size zone matrix (GLSZM) features, gray-level run-length matrix (GLRLM) features, and neighboring gray-tone difference matrix (NGTDM) features. This multi-dimensional approach generated 1,409 comprehensive features providing robust quantitative characterization of tumor morphology, intensity distribution, and textural complexity patterns.

Supplementary method 4: Computational Environment

Hardware Configuration: All experiments were conducted on a high-performance computing platform equipped with Intel Xeon Platinum 8488C processors running Windows 10 64-bit operating system. The computational

infrastructure included NVIDIA GeForce GTX 3080 Ti GPU acceleration and 128GB RAM to support intensive deep learning model training and radiomic feature extraction workflows.

Supplementary Table 1. Construction of M-DLR Score

Feature types	Feature name	Coefficient
Resnet18	resnet18_220.+02.	-0.007
	resnet18_324.+02.	0.286
Big modle	big_model_feature1017.+04.	0.254
	big_model_feature152.+00.	0.348
	big_model_feature139.+02.	-0.014
	big_model_feature314.+04.	0.248
	big_model_feature250.+04.	0.026
Peritumoral features	wavelet-LLH_glszm_SmallAreaEmphasis	0.709
	wavelet-LLH_glszm_SmallAreaLowGrayLevelEmphasis	0.666
3D radiomics	3d_rad_wavelet-HLH_glszm_SmallAreaHighGrayLevelEmphasis	0.324
Habitat features	3cluster_original_firstorder_Kurtosis_h2	0.016

Note: M-DLR Score: multi-dimensional imaging omics deep learning score. All the aforementioned features are derived from the arterial phase.

Supplementary Table 2: Clinical and Imaging Characteristics of Misclassified Cases

Characteristic	False Positive (n=33)	Correct G1 (n=134)	p-value	False Negative (n=40)	Correct G2/3 (n=200)	P value
Tumor size (cm)	3.45 ± 1.88	2.10 ± 1.44	<0.001	2.33 ± 2.43	4.22 ± 2.55	<0.001
TBS score	3.68 ± 1.80	2.46 ± 1.36	<0.001	2.62 ± 2.36	4.40 ± 2.47	<0.001
Cystic changes, n (%)	6/33 (18.2)	8/134 (6.0)	-	6/40 (15.0)	11/200 (5.5)	-
MD dilatation, n (%)	4/33 (12.1)	27/134 (20.1)	-	9/40 (22.5)	74/200 (37.0)	-
BD dilatation, n (%)	1/33 (3.0)	3/134 (2.2)	-	0/40 (0)	28/200 (14.0)	-
Tumor ≥3 cm, n (%)	17/33 (51.5)	23/134 (17.2)	-	-	-	-
Tumor ≤2 cm, n (%)	-	-	-	29/40 (72.5)	34/200 (17.0)	-

Note: TBS: tumor burden score; MD: main pancreatic duct; BD: bile duct
False positive cases represent G1 tumors misclassified as G2/3; False negative cases represent G2/3 tumors misclassified as G1. Statistical comparisons were performed using t-tests for continuous variables and chi-square tests for categorical variables where appropriate.

Supplementary Figure 1：Kaplan-Meier survival curves for progression-free survival stratified by M-DLR Score risk groups.

Patients were stratified into low-risk and high-risk groups using the optimal M-DLR Score cutoff value of 0.259. **(A)** Training set (n=230): Low-risk group (n=180) demonstrated superior progression-free survival compared to high-risk group (n=50). HR = 1.782 (95% CI: 1.168-2.721), log-rank p = 0.0066. Median PFS: 81.0 months vs. 53.9 months. **(B)** Validation set (n=92): Low-risk group (n=61) showed significantly longer progression-free survival than high-risk group (n=31). HR = 4.218 (95% CI: 1.973-9.017), log-rank p < 0.0001. Median PFS: not reached vs. 48.1 months. **(C)** External test set (n=54): Low-risk group (n=46) had better progression-free survival than high-risk group (n=8). HR = 2.643 (95% CI: 1.137-6.144), log-rank p = 0.0197. Median PFS: 55.0 months vs. 12.1 months. **(D)** Overall cohort (n=376): Low-risk group (n=287) demonstrated significantly superior progression-free survival compared to high-risk group (n=89). HR = 2.050 (95% CI: 1.484-2.833), log-rank p < 0.001. Blue lines represent low-risk patients, purple lines represent high-risk patients. Shaded areas indicate 95% confidence intervals. HR > 1 indicates higher hazard for progression in the high-risk group compared to the low-risk group. The M-DLR Score effectively stratified patients into distinct prognostic groups across all datasets, validating its clinical utility for risk assessment in pancreatic neuroendocrine tumors.

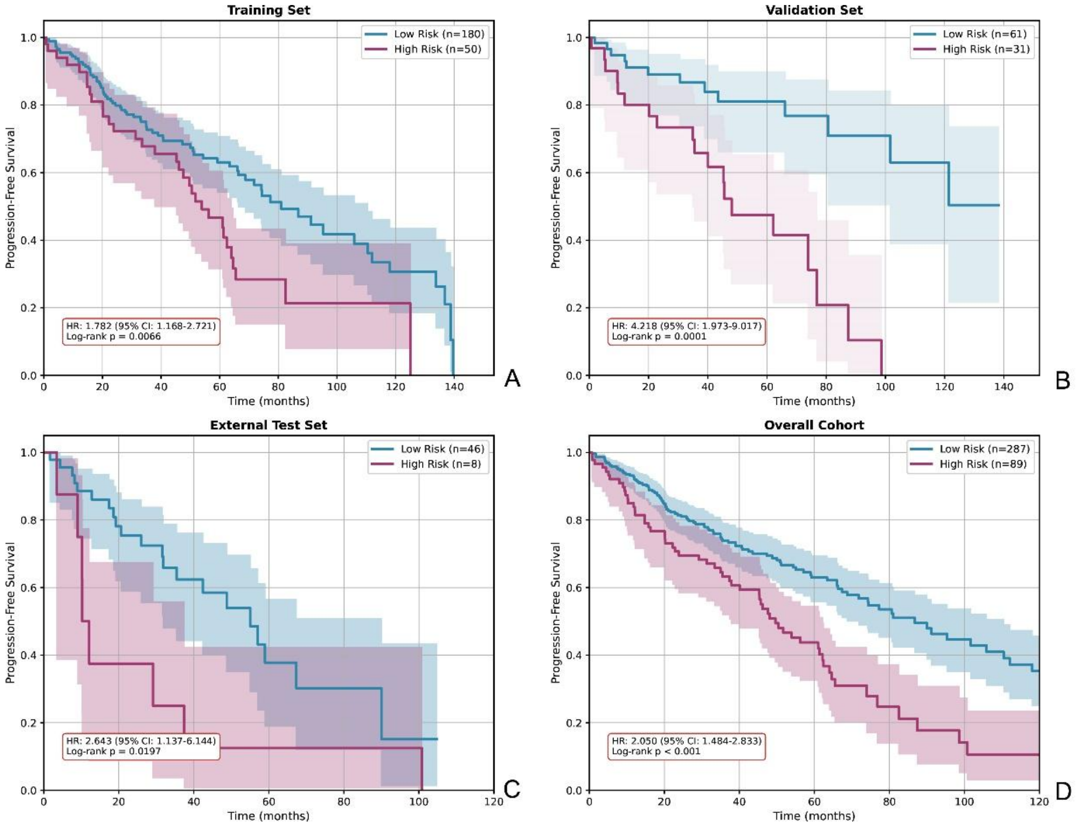
Training Set
Low Risk (n=180)
High Risk (n=50)
HR: 1.782 (95% CI: 1.168-2.721)
Log-rank p = 0.0066
Progression-Free Survival
Time (months)
A
Validation Set
Low Risk (n=61)
High Risk (n=31)
HR: 4.218 (95% CI: 1.973-9.017)
Log-rank p = 0.0001
Progression-Free Survival
Time (months)
B
External Test Set
Low Risk (n=46)
High Risk (n=8)
HR: 2.643 (95% CI: 1.137-6.144)
Log-rank p = 0.0197
Progression-Free Survival
Time (months)
C
Overall Cohort
Low Risk (n=287)
High Risk (n=89)
HR: 2.050 (95% CI: 1.484-2.833)
Log-rank p < 0.001
Progression-Free Survival
Time (months)
D

Supplementary Figure 2: Net Reclassification Improvement (NRI) analysis demonstrating PNET-PRISM's superior performance over the Clinical Model.

(A) Total NRI by dataset showing consistent positive improvement across all three datasets. PNET-PRISM achieved NRI values of 0.406 in the training set, 0.127 in the validation set, and 0.318 in the external test set, indicating clinically meaningful enhancement in patient risk stratification. **(B)** Area under the curve (AUC) comparison between the Clinical Model (red bars) and PNET-PRISM (blue bars) across all datasets. PNET-PRISM demonstrated superior discrimination performance with substantial AUC improvements: +0.152 in training, +0.142 in validation, and +0.176 in external test sets. **(C)** Reclassification matrix for the training set illustrating patient movement between prediction categories when transitioning from Clinical Model to PNET-PRISM. Green cells represent improved reclassification (16 G2/3 patients correctly upgraded from G1 to G2/3 prediction, and 32 G1 patients correctly downgraded from G2/3 to G1 prediction), while red cells represent worsened reclassification (6 G2/3 patients incorrectly downgraded and 1 G1 patient incorrectly upgraded). **(D)** Summary statistics for all three datasets showing total NRI, AUC improvement, and number of patients reclassified. The consistently positive NRI values across all datasets demonstrate PNET-PRISM's robust advantage over the Clinical Model in patient risk stratification, with the greatest improvements observed in the training (NRI=0.406) and external test sets (NRI=0.318).

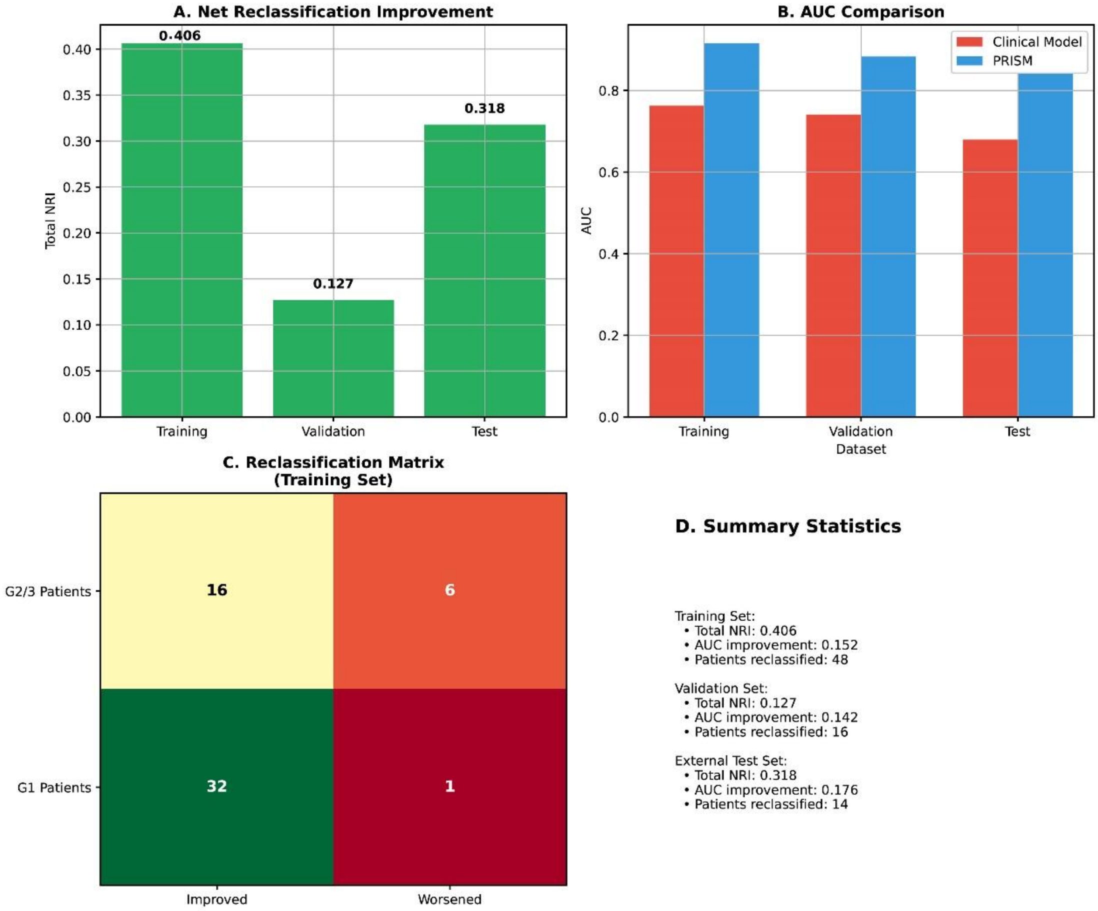
A. Net Reclassification Improvement
Total NRI
0.406
0.127
0.318
0.00
0.05
0.10
0.15
0.20
0.25
0.30
0.35
0.40
Training
Validation
Test
B. AUC Comparison
Clinical Model
PRISM
AUC
0.0
0.2
0.4
0.6
0.8
Training
Validation
Test
Dataset
C. Reclassification Matrix
(Training Set)
G2/3 Patients
G1 Patients
16
6
32
1
Improved
Worsened
D. Summary Statistics
Training Set:
• Total NRI: 0.406
• AUC improvement: 0.152
• Patients reclassified: 48
Validation Set:
• Total NRI: 0.127
• AUC improvement: 0.142
• Patients reclassified: 16
External Test Set:
• Total NRI: 0.318
• AUC improvement: 0.176
• Patients reclassified: 14

Supplementary Figure 3: Representative false positive cases: G1 PNETs misclassified as G2/3 by the PNET-PRISM model

(A-D) Case 1 (49-year-old male, 8.0 cm pancreatic tail tumor, pathologically confirmed G1): **(A)** Arterial-phase CT demonstrates cystic degeneration. **(B)** Automated segmentation (red: tumor; green: pancreatic parenchyma). **(C)** Habitat analysis revealing intratumoral heterogeneity patterns. PNET-PRISM prediction: 96.7% probability of G2/G3 (false positive) (D) Overall G2/G3 probability distribution (pie chart: 96.7%)

(E-H) Case 2 (55-year-old female, 9.7 cm pancreatic head tumor, pathologically confirmed G1): **(E)** Arterial-phase CT shows markedly dilated main pancreatic duct. **(F)** Multi-structure segmentation (red: tumor; green: pancreatic parenchyma; blue: pancreatic duct). **(G)** Habitat analysis with periductal microenvironment features. PNET-PRISM prediction: 64.8% probability of G2/G3 (false positive) **(H)** Corresponding probability pie chart

(I-L) Case 3 (66-year-old male, 1.7 cm pancreatic head tumor, pathologically confirmed G1): **(I)** Arterial-phase CT shows markedly dilated main pancreatic duct. **(J)** Multi-structure segmentation (red: tumor; green: pancreatic parenchyma; blue: pancreatic duct). **(K)** Habitat analysis with periductal microenvironment features. PNET-PRISM prediction: 98.7% probability of G2/G3 (false positive) **(L)** Corresponding probability pie chart

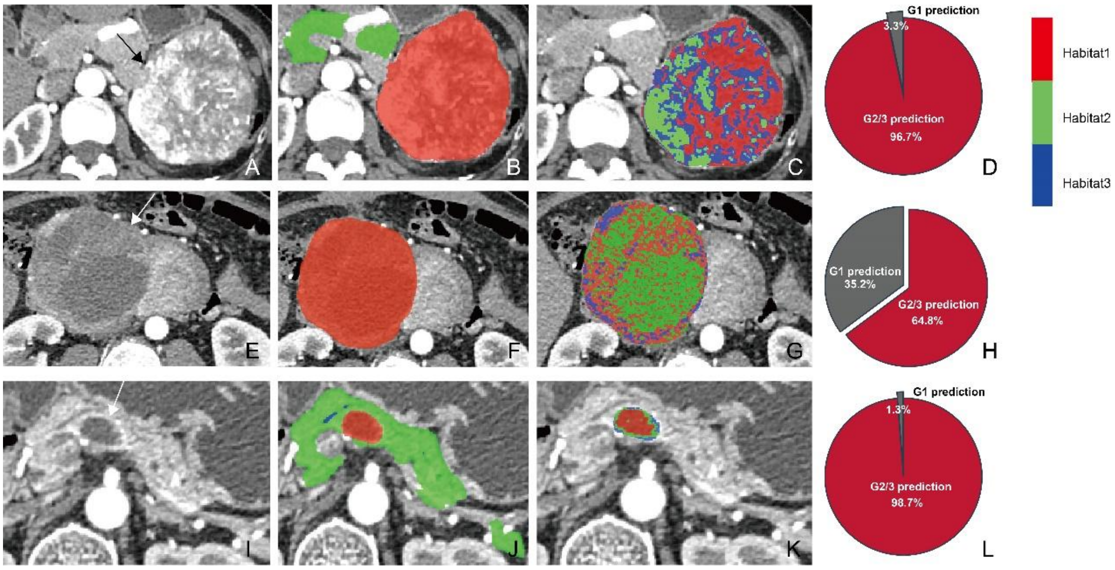
A
B
C
G1 prediction
3.3%
G2/3 prediction
96.7%
D
Habitat1
Habitat2
Habitat3
E
F
G
G1 prediction
35.2%
G2/3 prediction
64.8%
H
I
J
K
G1 prediction
1.3%
G2/3 prediction
98.7%
L

Supplementary Figure 4. Representative false negative cases: G2/3 PNETs misclassified as G1 by the PNET-PRISM model

(A-D) Case 1 (62-year-old female, 1.8 cm pancreatic head tumor, pathologically confirmed G2): **(A)** Arterial-phase CT demonstrates homogeneous hyperenhancement mimicking G1 tumor. **(B)** Automated segmentation (red: tumor; green: pancreatic parenchyma). **(C)** Habitat analysis reveals limited intratumoral heterogeneity. PNET-PRISM prediction: 11.9% probability of G2/G3 (false negative). **(D)** Overall G2/G3 probability distribution (pie chart: 11.9%)

(E-H) Case 2 (53-year-old male, 1.5 cm pancreatic tail tumor, pathologically confirmed G2): **(E)** Arterial-phase CT shows minimal enhancement suggesting low metabolic activity. (F) Tumor segmentation. **(G)** Habitat analysis demonstrates subthreshold heterogeneity. PNET-PRISM prediction: 62.9% probability of G2/G3 (false negative). **(H)** Corresponding probability pie chart

(I-L) Case 3 (58-year-old female, 2.1 cm pancreatic head tumor, pathologically confirmed G3): **(I)** Arterial-phase CT with ill-defined hypo-enhancement. **(J)** Tumor segmentation. **(K)** Habitat analysis shows insufficient heterogeneity for high-grade classification. PNET-PRISM prediction: 3.7% probability of G2/G3 (false negative). **(L)** Corresponding probability pie chart

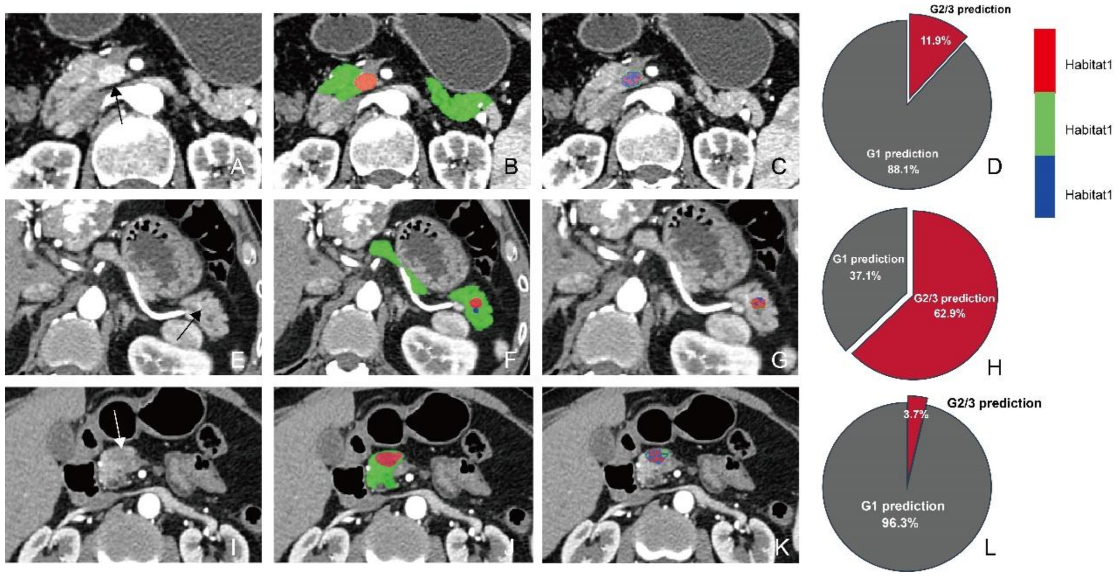
A
B
C
D
G2/3 prediction
11.9%
G1 prediction
88.1%
Habitat1
Habitat1
Habitat1
E
F
G
H
G1 prediction
37.1%
G2/3 prediction
62.9%
I
J
K
L
G2/3 prediction
3.7%
G1 prediction
96.3%

Supplementary Figure 5: Clinical decision-making flowchart for PNET-PRISM (Pancreatic Radiomics Integrated Scoring Model) implementation in PNET management.

The flowchart illustrates the comprehensive clinical workflow for implementing PNET-PRISM in preoperative PNET grading and treatment decision-making. Starting from initial PNET identification on CT imaging, the algorithm guides clinicians through standardized image acquisition, automated radiomics analysis, and risk-stratified management strategies. Key decision points include tumor size thresholds (2cm and 3cm), presence of cystic changes, secondary signs (bile duct/pancreatic duct dilatation), and M-DLR Score stratification (cutoff: 0.259). For predicted G1 tumors, management ranges from high-confidence surveillance (≤2cm, no cystic changes) to EUS-FNA confirmation (>2cm or cystic features). For predicted G2/3 tumors, strategies include high-confidence surgical resection (≥3cm with secondary signs) or multidisciplinary discussion for borderline cases. The model demonstrates particular clinical utility in cases where EUS-FNA is contraindicated or yields inadequate tissue (52% accuracy in failed EUS cases). PNET-PRISM: Pancreatic Radiomics Integrated Scoring Model; M-DLR: Multidimensional Deep Learning Radiomics; EUS-FNA: Endoscopic ultrasound-guided fine needle aspiration; MDT: Multidisciplinary team; PNET: Pancreatic neuroendocrine tumor.

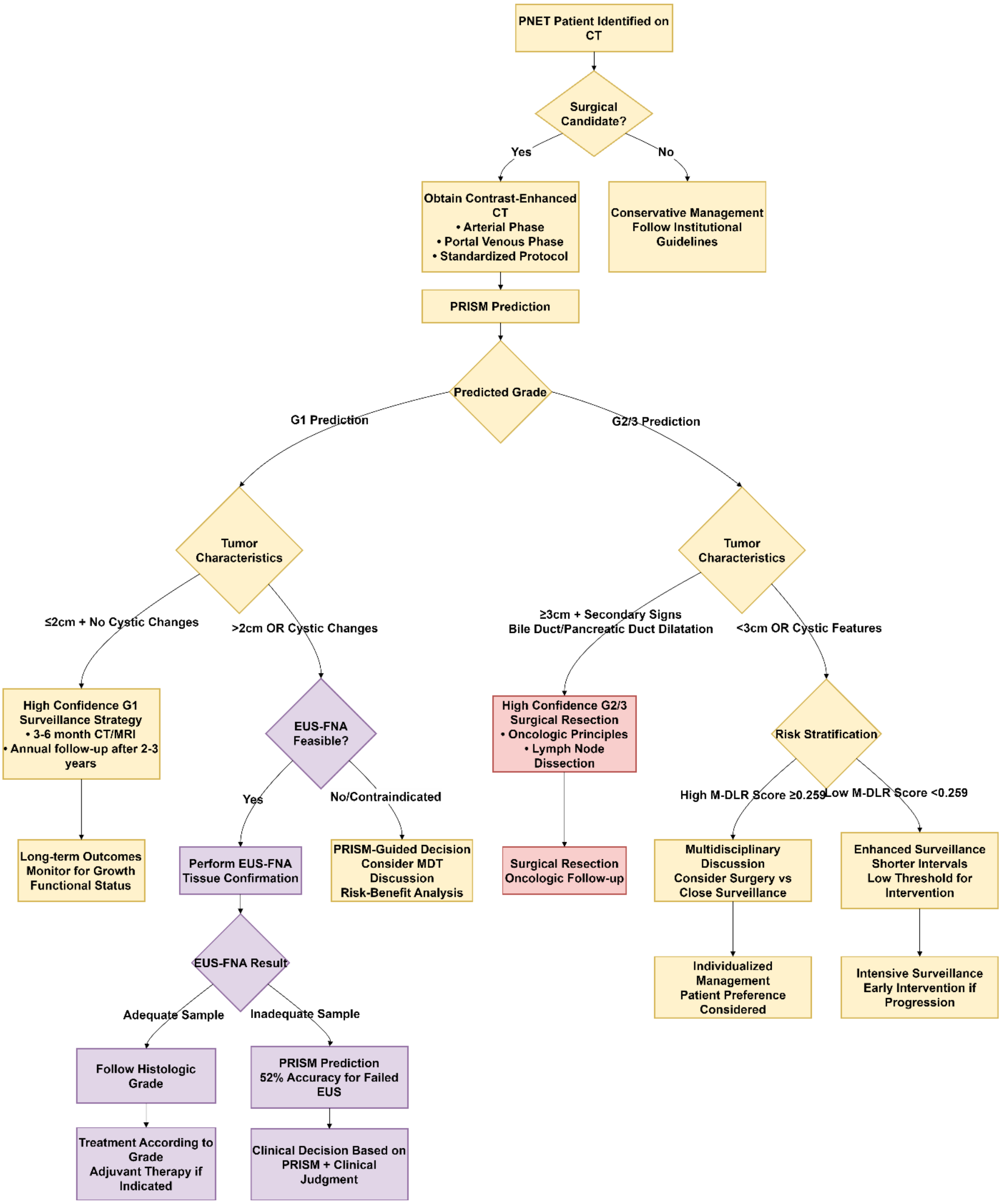
PNET Patient Identified on CT
Surgical Candidate?
Yes
No
Obtain Contrast-Enhanced CT
• Arterial Phase
• Portal Venous Phase
• Standardized Protocol
Conservative Management
Follow Institutional Guidelines
PRISM Prediction
Predicted Grade
G1 Prediction
G2/3 Prediction
Tumor Characteristics
Tumor Characteristics
≤2cm + No Cystic Changes
>2cm OR Cystic Changes
≥3cm + Secondary Signs
Bile Duct/Pancreatic Duct Dilatation
<3cm OR Cystic Features
High Confidence G1
Surveillance Strategy
• 3-6 month CT/MRI
• Annual follow-up after 2-3 years
EUS-FNA Feasible?
High Confidence G2/3
Surgical Resection
• Oncologic Principles
• Lymph Node Dissection
Risk Stratification
Yes
No/Contraindicated
High M-DLR Score ≥0.259
Low M-DLR Score <0.259
Long-term Outcomes
Monitor for Growth
Functional Status
Perform EUS-FNA
Tissue Confirmation
PRISM-Guided Decision
Consider MDT Discussion
Risk-Benefit Analysis
Surgical Resection
Oncologic Follow-up
Multidisciplinary Discussion
Consider Surgery vs Close Surveillance
Enhanced Surveillance
Shorter Intervals
Low Threshold for Intervention
EUS-FNA Result
Individualized Management
Patient Preference Considered
Intensive Surveillance
Early Intervention if Progression
Adequate Sample
Inadequate Sample
Follow Histologic Grade
PRISM Prediction
52% Accuracy for Failed EUS
Treatment According to Grade
Adjuvant Therapy if Indicated
Clinical Decision Based on PRISM + Clinical Judgment

References

1. van Griethuysen JJM, Fedorov A, Parmar C, et al. Computational Radiomics System to Decode the Radiographic Phenotype. *Cancer Res* 2017; 77:e104-e107
